# Cardiac CT in criss-cross heart

**DOI:** 10.1259/bjrcr.20200096

**Published:** 2020-10-15

**Authors:** Harsumeet Singh Sidhu, Munish Guleria

**Affiliations:** 1 Department of Radiodiagnosis, ABVIMS and Dr RML Hospital, New Delhi, India

## Abstract

A criss-cross heart is an uncommon congenital rotational anomaly. It accounts for less than 0.1% of all congenital heart defects. The anomaly is characterized by crossing of the atrioventricular connections caused by rotation of the heart about its long axis. It is commonly associated with diverse cardiac defects. Cardiac CT imaging of criss-cross heart is sparse. We present a case of 1-year-old child with chief complaints of bluish discoloration of the body and fast breathing. Cardiac CT revealed atrial situs solitus, criss-cross-atrioventricular connections, atrioventricular discordance, double outlet right ventricle and dextro-malposed great arteries (Van Praagh S,D,D).

## Introduction

Congenital heart malformations occur in around eight cases per 1000 births. Criss-cross-heart is an uncommon anomaly, not exceeding 8 per 1 000 000 births and accounting for less than 0.1% of all congenital heart malformations.^1^ The atrioventricular structures are normally parallel to each other whereas angulated by as much as 90 degrees in a criss-cross heart.^2–5^ Crossing of the systemic and pulmonary venous inflows occurs separately at the atrioventricular level in this congenital rotational anomaly. The crossed atrioventricular connections can be concordant or discordant. The ventricles are seen in superior-inferior relationship with the right ventricle residing on top of the left ventricle.

## Case report

A 1-year-old male child presented to the outpatient department with complaints of bluish discoloration of the body and fast breathing for last 1 month. Bluish discoloration was sudden in onset involving nail beds and lips. It was associated with fast breathing which was sudden in onset, progressive and increased during evening. On examination, he presented severe dyspnea with nasal flaring, subcostal and intercostal indrawing and retraction of sternal notch. He was cyanosed and afebrile. His heart rate was 110 bpm and respiratory rate 45 bpm. Blood pressure was recorded at 85/35 mm Hg and oxygen saturation 70% in room air. All peripheral pulses were palpable. Cardiac examination revealed normal first and second heart sounds and a systolic murmur at the left upper parasternal area. The child was managed with prostaglandin infusion which mildly improved his condition. The child had normal term vaginal delivery with birth weight of 2.6 kgs. He cried immediately after birth. The pregnancy was uneventful. No family history of congenital heart disease was present.

The initial transthoracic echocardiographic assessment revealed: criss-cross heart with discordant atrioventricular connections, double outlet right ventricle, large ventricular septal defect, and subvalvular pulmonary stenosis.

CT was done on 128 slice Dual Energy CT scanner (SOMATOM Definition Flash; Siemens, Erlangen, Germany). CT data were obtained with the following parameters: 3 mm slice thickness, 3 mm increment, 0.28 s rotation time, 0.38 pitch and reconstructed images of 0.6 mm slice thickness and 0.6 mm increment. A low effective radiation dose of 0.2 milli Sieverts was given to the patient. A volume of 2 ml kg^−1^ bodyweight of non-ionic iodinated contrast agent was administered through IV cannula at a rate of 2 ml s^−1^ via a power injector. Care bolus tracking technique was employed with the region of interest placed within the descending thoracic aorta, at the level of the carina and scan threshold set at 100 Hounsefield units (HU). Contrast saline solution of 50% dilution was utilized in bolus chasing technique to improve contrast within the cardiac chambers. ECG synchronized retrospective gating technique was employed for cardiovascular evaluation.

**Figure 1.**
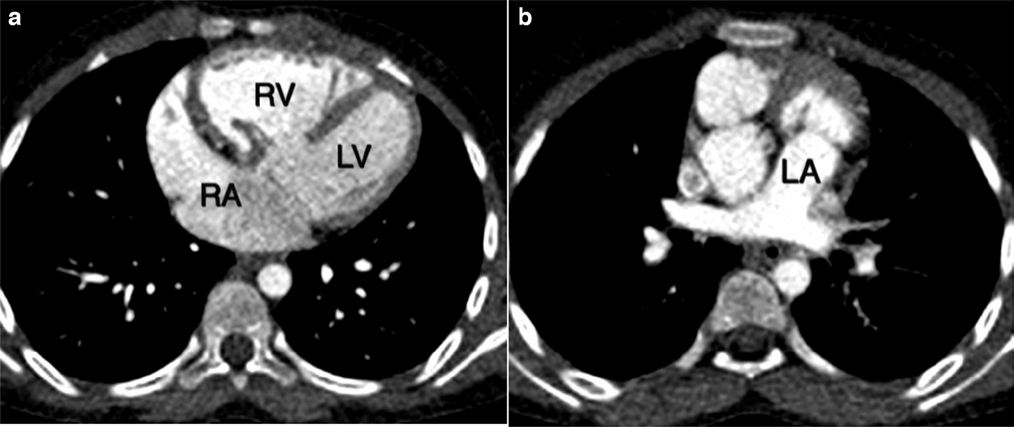
Axial image at midventricular level showed morphologic right atrium on right side in (a) and axial image superior to midventricular level showed morphologic left atrium on left side in (b) consistent with atrial situs solitus. Morphological right ventricle (RV) was noted on right side-of morphological left ventricle (LV) in (a) consistent with dextro-loop topology. LA: Left atrium, LV: Left ventricle, RA: Right atrium, RV: Right ventricle.

**Figure 2.**
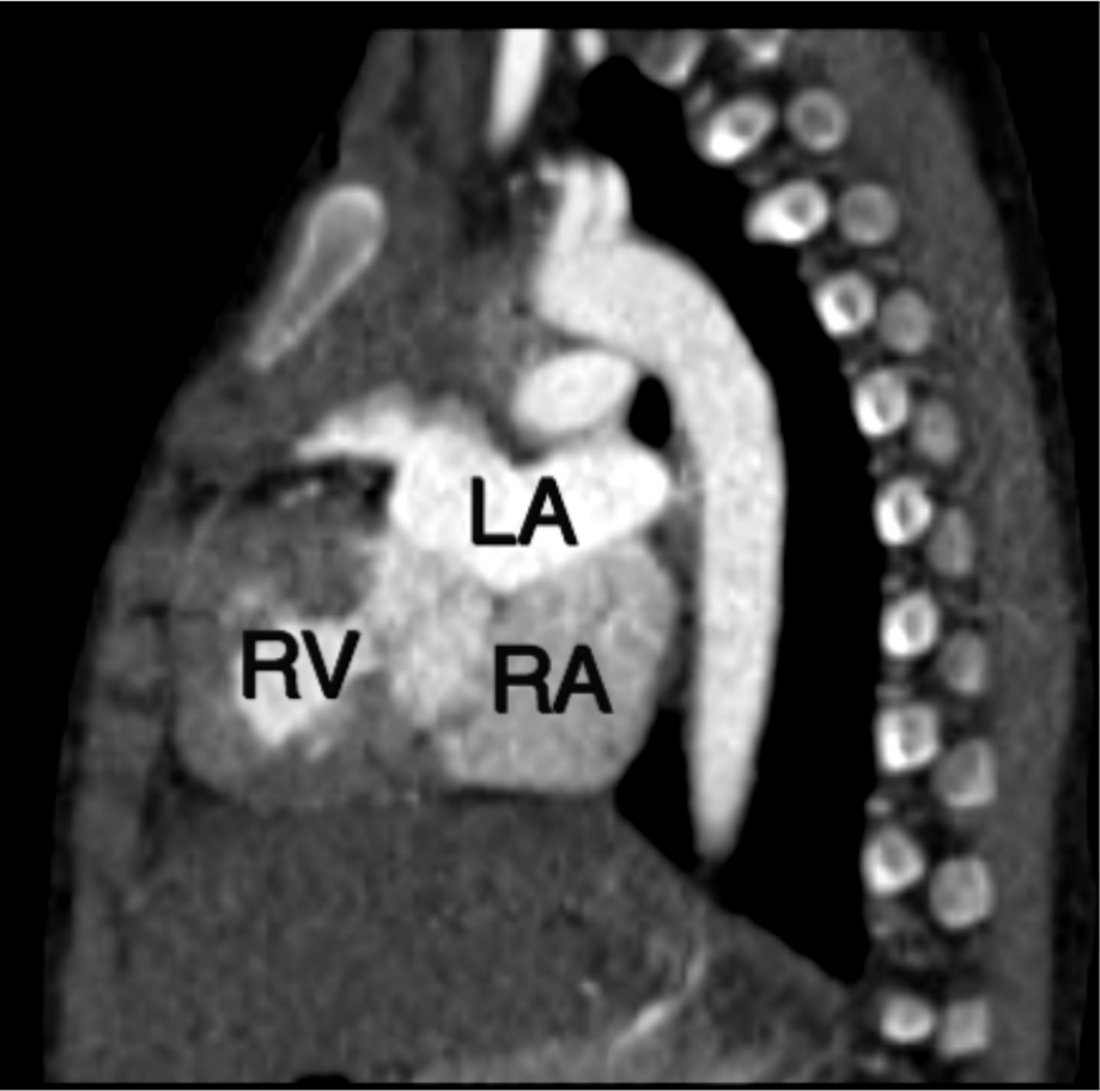
Sagittal image showed left atrium residing on top of right atrium. LA: Left atrium, RA: Right atrium, RV: Right ventricle.

**Figure 3.**
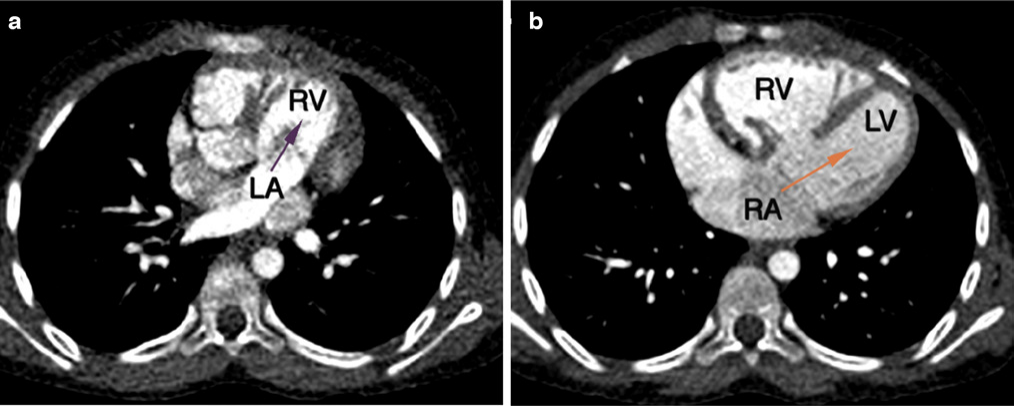
(a) Axial image superior to midventricular level showed left atrium draining into right ventricle. (b) Axial image at midventricular level showed right atrium draining into left ventricle consistent with atrioventricular discordance and criss-cross appearance at atrioventricular level. LA: Left atrium, LV: Left ventricle, RA: Right atrium, RV: Right ventricle.

**Figure 4.**
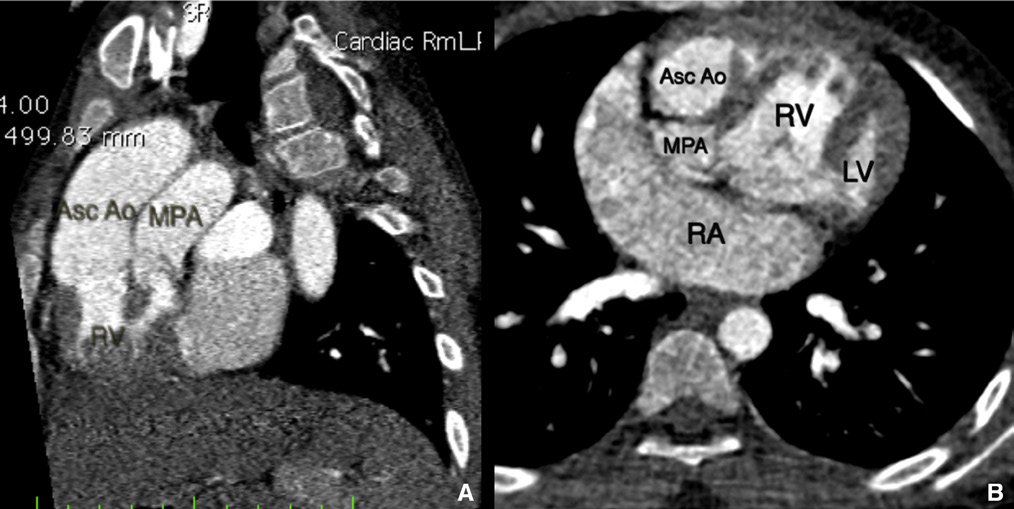
(a) Sagittal oblique image showed both ascending aorta and main pulmonary artery arising from the right ventricle s/o double outlet right ventricle. (b) Axial image at the level of right ventricular outflow tract showed aorta anterior and slightly to the right of main pulmonary artery s/o dextro-malposed great arteries. Asc: Aoascending aorta, LV: Left ventricle, MPA: Main pulmonary artery, RA: right atrium, RV: Right ventricl.

**Figure 5.**
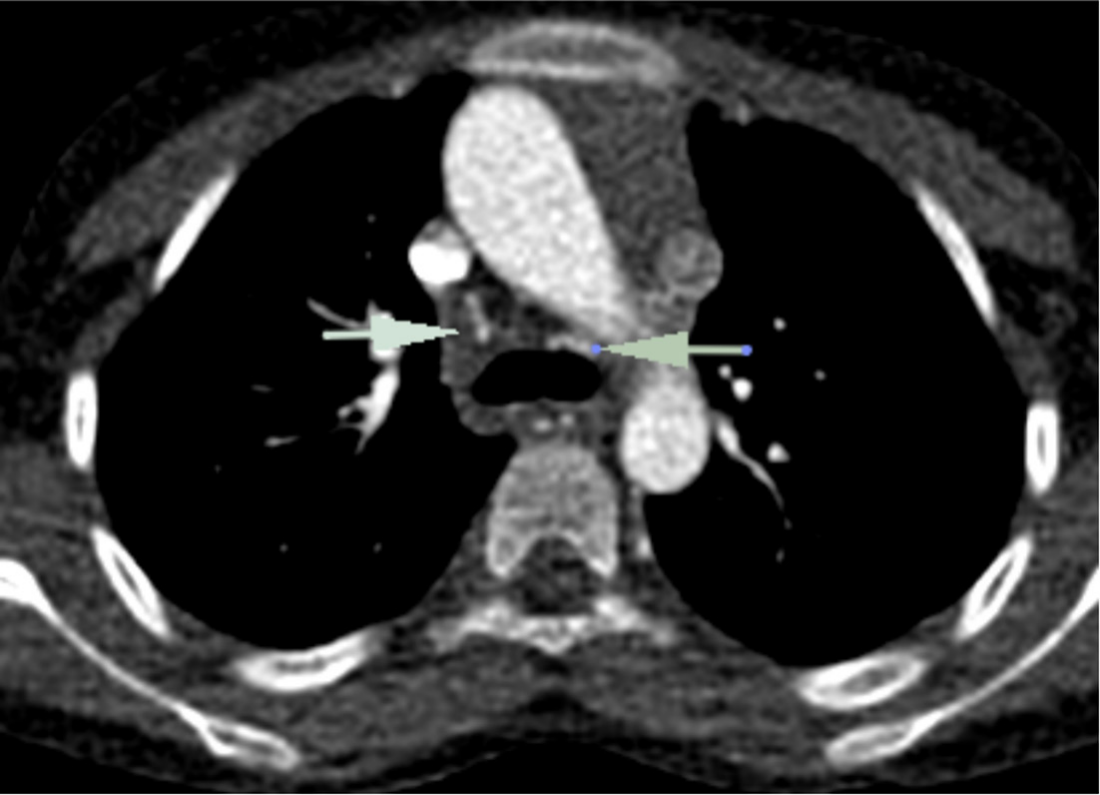
Axial image at the level of arch of aorta showed aortopulmonary collaterals (arrows). These collaterals had origin from right subclavian artery and descending thoracic aorta.

Cardiac CT revealed atria in solitus position with morphological right atrium on right side and morphologic left atrium on left side (Figure 1a and b). Dextro-loop topology was noted with morphological right ventricle to the right of morphological left ventricle (Figure 1a). Left atrium and right atrium were noted in supero-inferior relationship, respectively (Figure 2). Crossing axes of atrioventricular connections and atrioventricular discordance showing the right atrium draining into morphological left ventricle and left atrium draining into morphological right ventricle were seen (Figure 3a and b). Both aorta and main pulmonary artery were noted arising from right ventricle s/o double outlet right ventricle (Figure 4a). Aortic was located anterior and slightly to the right of main pulmonary artery s/o dextro-malposed great arteries (Figure 4b). Significant subvalvular pulmonary stenosis was noted. Aortopulmonary collaterals were noted (Figure 5). These were seen arising from right subclavian artery and descending thoracic aorta. Large inlet ventricular septal defect with extension into outlet beneath pulmonary valve was noted. Double superior vena cava was present. Left superior vena cava drained into right atrium through coronary sinus. The above described case represented atrial situs solitus, criss-cross-atrioventricular connections with atrioventricular discordance, double outlet right ventricle, dextro-malposed great arteries, ventricular septal defect and subvalvular pulmonary stenosis. Fontan procedure was performed. The definitive repair included closure of ventricular septal defect, ligation of the proximal pulmonary trunk and a conduit was placed between the left ventricle and the distal pulmonary trunk. Post-operative period was uneventful. The patient’s condition was satisfactory at discharge.

## Discussion

Criss-cross heart is an extremely rare complex congenital heart malformation. It was first reported by Lev and Rowlatt in 1961.^6^ Criss-cross heart was termed by Anderson et al as a cardiac anomaly characterized by crossing of the systemic and pulmonary venous inflows at the atrioventricular level without mixing.^3^ Many criss-cross heart cases with situs solitus, situs inversus or isomerism were described in the later years.^5,7^

In a normal heart, the axes of atrioventricular connections are parallel, however in a criss-cross heart there is intersection of these axes. The spatial position of base of the heart remains unchanged whereas the ventricles twist along their longitudinal axis. This rotation leads to crossing of the inflows through the atrioventricular valves. As a result, each atrium is positioned towards the contralateral ventricle. The criss-cross heart is associated with diverse cardiac defects. These include ventricular septal defect, transposition of great vessels, double outlet right ventricle, right ventriclular hypoplasia, tricuspid hypoplasia and pulmonary stenosis. Less frequently seen are mitral or tricuspid valvular straddling, mitral stenosis, subaortic stenosis and aortic arch anomalies.^8–10^

Few cases of criss-cross heart showing atrioventricular discordance and double outlet right ventricle have been reported, mostly at postmortem^11,12^ and seldom during life.^13^ Podzolkov et al reported a case of criss-cross heart with atrioventricular discordance, double outlet right ventricle, dextro-malposed great vessels, ventricular septal defect, subvalvular and valvular pulmonary stenosis with hypoplastic annulus and persistent left superior vena cava draining into the coronary sinus.^14^ In another study, Oliveira et al^15^ reported a case showing of criss-cross atrioventricular connections with atrioventricular discordance and double outlet right ventricle. The case described in our report also revealed criss-cross atrioventricular connections, atrioventricular discordance, double outlet right ventricle, dextro-malposed great vessels, ventricular septal defect and subvalvular pulmonary stenosis. In such cases, the surgical correction includes: (a) ventricular septal defect is closed through the arterial morphologic right ventricle or venous morphologic left ventricle (b) pulmonary trunk is ligated and (c) a conduit is placed to connect the venous ventricle to the pulmonary trunk. Thus, the definite repair of such cardiac anomaly with criss-cross-atrioventricular connections with double outlet right ventricle and pulmonary stenosis is according to the Fontan procedure.^14^

Majority of criss-cross-heart cases have been assessed by echocardiography^16,17^ and a few by MRI^18,19^ as reported in the literature. The CT literature on this anomaly is sparse. A single study of five cases of criss-cross-heart was published in which the patients were imaged with old generation CT scanner.^20^ Nowadays, the cardiac study is performed with very low radiation doses with the newer generations of CT scanners. The newer CT scanners are also very quick in acquiring images, thus useful in imaging of children requiring no sedation and with fewer motion artifacts. The obtained images are of high quality and can be reviewed in multiple reconstruction planes. Thus, CT imaging is useful in the pre-operative evaluation of this complex congenital anomaly and aid in suitable surgical intervention.

## Conclusion

Criss-cross heart is an uncommon congenital cardiac malformation. As it is commonly associated with diverse cardiac defects, this rotational anomaly requires early diagnosis so that appropriate surgical management can be provided to achieve a good functional outcome.

## Learning points

- Criss-cross heart is an uncommon congenital anomaly characterized by crossing of the systemic and pulmonary venous inflows separately at the atrioventricular level.
- In case of inadequate pulmonary blood supply, initial management with prostaglandins is indicated followed by systemic to pulmonary shunting.
- Fontan type operation is performed in criss-cross heart with atrioventricular discordance and double outlet right ventricle.
- Cardiac CT provides an ideal alternative to cardiac catheterization and echocardiography in the pre-operative evaluation of this complex congenital anomaly.
